# Use of Anticoagulation Therapy in Patients With Perioperative Atrial Fibrillation After Cardiac Surgery: A Systematic Review and Meta-analysis

**DOI:** 10.1016/j.cjco.2022.06.003

**Published:** 2022-06-10

**Authors:** Michael Ke Wang, Rachel Heo, Pascal Meyre, Louis Park, Steffen Blum, William F. McIntyre, Emilie Belley-Côté, Lauren Birchenough, Kiven Vuong, Jeff S. Healey, P.J. Devereaux, André Lamy, David Conen

**Affiliations:** a Department of Medicine, McMaster University, Hamilton, Ontario, Canada; b Population Health Research Institute, McMaster University, Hamilton, Ontario, Canada; c Department of Health Research Methods, Evidence & Impact, McMaster University, Hamilton, Ontario, Canada; d Michael G. DeGroote School of Medicine, McMaster University, Hamilton, Ontario, Canada; e Division of Cardiology and Cardiovascular Research Institute Basel, University Hospital Basel, Basel, Switzerland; f Faculty of Health Sciences, McMaster University, Hamilton, Ontario, Canada; g Faculty of Sciences, Western University, London, Ontario, Canada; h Department of Surgery, McMaster University, Hamilton, Ontario, Canada

## Abstract

**Background:**

Perioperative atrial fibrillation (POAF) after cardiac surgery is associated with an increased risk of stroke. However, the efficacy and safety of using anticoagulation therapy in this population are unknown.

**Methods:**

We performed a systematic review and meta-analysis of studies comparing use of anticoagulation therapy vs no anticoagulation therapy in patients with POAF after cardiac surgery. Outcomes included arterial thromboembolism (ie, stroke ± systemic embolism) and bleeding. Data were pooled using fixed-effects models. We reported summary risk ratios (RRs) for studies with multivariable adjustment and estimated absolute risk differences with 95% confidence intervals (CIs).

**Results:**

Nine observational studies met eligibility criteria. No randomized trials were identified. Of the 254,200 POAF patients included, 27.3% received anticoagulation. Six studies reported outcomes after long-term follow-up (median 5.0 years; range 4.2-10.0). The risk of arterial thromboembolism was lower in patients receiving anticoagulation therapy (RR 0.83; 95% CI, 0.69-0.99; I^2^ = 57%; *P* = 0.04; 6 studies). The estimated short-term and long-term absolute risk reductions in arterial thromboembolism with use of anticoagulation therapy were 0.8% (95% CI, 0.4-1.4) and 2 events per 1000 person-years (95% CI, 0-4), respectively. The risk of bleeding was higher in patients receiving anticoagulation therapy (RR 3.22; 95% CI, 2.82-3.68; I^2^ = 98%; *P* < 0.001; 2 studies). The estimated short-term and long-term absolute risk increases in bleeding with use of anticoagulation therapy were 0.5% (95% CI, 0.4-0.6) and 42 events per 1000 person-years (95% CI, 35-51), respectively.

**Conclusions:**

Use of anticoagulation therapy is associated with a small reduction in the risk of arterial thromboembolism, but also an increased risk of bleeding. Randomized controlled trials are needed to address this issue.

## Résumé

**Introduction:**

La fibrillation auriculaire périopératoire (FAPO) après l’intervention chirurgicale au cœur est associée à une augmentation du risque d’accident vasculaire cérébral (AVC). Toutefois, on ne connaît pas l’efficacité et l’innocuité de la l’anticoagulothérapie de cette population.

**Méthodes:**

Nous avons réalisé une revue systématique et une méta-analyse d’études qui comparaient l’utilisation de l’anticoagulothérapie *vs* l’absence d’anticoagulothérapie chez les patients atteints de FAPO après l’intervention chirurgicale au cœur. Les résultats étaient notamment la thromboembolie artérielle (c.-à-d. l'AVC ± l’embolie systémique) et les hémorragies. Nous avons regroupé les données à l’aide de modèles à effets fixes. Nous avons rapporté les risques relatifs (RR) sommaires d’études avec l’ajustement multivarié et l’estimation des différences du risque absolu avec des intervalles de confiance (IC) à 95 %.

**Résultats:**

Neuf études observationnelles répondaient aux critères d’admissibilité. Aucun essai à répartition aléatoire n’a été trouvé. Parmi les 254 200 patients atteints de FAPO sélectionnés, 27,3 % avaient reçu une anticoagulation. Six études révélaient des résultats après le suivi à long terme (médiane 5,0 ans ; fourchette 4,2-10,0). Le risque de thromboembolie artérielle était plus faible chez les patients qui avaient reçu une anticoagulothérapie (RR 0,83 ; IC à 95 %, 0,69-0,99 ; I^2^ = 57 % ; *P* = 0,04 ; six études). Les estimations de réduction du risque absolu à court terme et à long terme lors de thromboembolie artérielle avec l’utilisation de l’anticoagulothérapie étaient respectivement de 0,8 % (IC à 95 %, 0,4-1,4) et de deux événements par 1000 personnes-années (IC à 95 %, 0-4). Le risque d’hémorragie était plus élevé chez les patients qui avaient reçu une anticoagulothérapie (RR 3,22 ; IC à 95 %, 2,82-3,68 ; I^2^ = 98 % ; *P* < 0,001 ; deux études). Les estimations d’augmentation du risque absolu à court terme et à long terme des hémorragies avec l’utilisation de l’anticoagulothérapie étaient respectivement de 0,5 % (IC à 95 %, 0,4-0,6) et de 42 événements par 1000 personnes-années (IC à 95 %, 35-51).

**Conclusions:**

L’utilisation de l’anticoagulothérapie est associée à une réduction minime du risque de thromboembolie artérielle, mais aussi à une augmentation du risque d’hémorragie. Des essais cliniques à répartition aléatoire sont nécessaires pour aborder cette question.

Perioperative atrial fibrillation (POAF) is the most common arrhythmia encountered after cardiac surgery, with the estimated incidence of new-onset POAF ranging between 24% and 49%.^1^ Although POAF has been viewed as a benign and self-limited phenomenon, a growing body of evidence suggests that POAF is associated with an increased short-term and long-term risk of stroke and death.^1^, ^2^, ^3^

Oral anticoagulation therapy is highly effective for the long-term prevention of ischemic stroke and systemic embolism in patients with nonsurgical atrial fibrillation (AF).^4^ However, the absolute risk of these events may be lower in patients with POAF.^1^ The efficacy and safety of anticoagulation in patients with POAF after cardiac surgery are currently unclear. Moreover, given that the incidences of adverse outcomes after surgery change over time, the absolute risks and benefits of short-term and long-term use of anticoagulation therapy after POAF need to be considered separately.

To address these knowledge gaps, we performed a systematic review and meta-analysis on studies evaluating the effects of anticoagulation therapy in patients with POAF after cardiac surgery.

## Methods

We reported this systematic review and meta-analysis according to the **M**eta-analysis **o**f **O**bservational **S**tudies in **E**pidemiology (MOOSE) reporting guidelines.^5^ We registered the study protocol with the International Prospective Register of Systematic Reviews (PROSPERO) (CRD42021257115).

### Search methods

We identified relevant studies through a systematic literature search of the MEDLINE (National Library of Medicine), Excerpta Medica database (Embase; Elsevier), and Cochrane Central Register of Controlled Trials (CENTRAL) databases covering the period from the time of database inception until January 25, 2022. Eligible studies were identified using a search strategy combining keywords and terms related to surgery, atrial fibrillation, and anticoagulation (Supplemental Methods S1). We identified additional articles by reviewing reference lists from relevant studies and consulting with experts in the field.

### Study selection

We considered cohort studies, case-control studies, and randomized controlled trials to be eligible for inclusion. Studies were included if they met the following criteria: (i) they included only patients undergoing cardiac surgery; (ii) they reported 1 or more outcomes in patients with vs without anticoagulation use after surgery; (iii) they had ≥ 100 participants with POAF; and (iv) they included patients ≥ 18 years of age. We excluded the following: (i) studies in which anticoagulation therapy was routinely prescribed after surgery (eg, mechanical valve replacement); (ii) studies not published as full-text articles (ie, meeting abstracts); and (iii) observational studies that reported study outcomes without multivariable adjustment. Studies including patients with preoperative AF were considered eligible. Studies were not excluded on the basis of the language of publication. Title and abstract screening and full-text review were conducted independently and in duplicate, with discrepancies resolved through consensus or by a third independent reviewer.

### Outcome assessment

We defined the use of anticoagulation therapy as prescription of any anticoagulation drug formulation (ie, oral, intravenous, or subcutaneous) after the diagnosis of POAF, at doses considered to be therapeutic for the prevention of arterial thromboembolism in patients with nonsurgical AF.

The main study outcomes were arterial thromboembolism and bleeding. We defined arterial thromboembolism as stroke with or without systemic embolism. Acceptable definitions of stroke included any stroke, ischemic stroke, and embolic stroke. Any established definition of bleeding was considered acceptable. Other outcomes included all-cause mortality, myocardial infarction, and venous thromboembolism. Short-term events were defined as events occurring within the first 3 months after surgery, and long-term events were defined as events occurring more than 3 months after surgery.

### Data extraction

Data extraction was performed independently and in duplicate using standardized forms. Information was collected on the study design, sample size, types of surgical procedures, baseline demographics, concomitant medication use (including antiplatelet drug use), study definitions (ie, POAF, anticoagulation therapy use, outcomes), number of POAF patients using anticoagulation therapy, reported associations between anticoagulation therapy use and outcomes, and covariates used for multivariable adjustment. We contacted the corresponding authors of eligible studies to obtain missing and unpublished data.

### Assessment of risk of bias and certainty of the evidence

The Risk **o**f Bias in Non-randomised Studies - of Interventions (ROBINS)-I tool was used to assess the risk of bias for nonrandomized studies.^6^ The tool assesses 7 bias domains and views each study as a hypothetical randomized controlled trial. Risk of bias assessment was completed independently and in duplicate. Disagreements were resolved through consensus, consistent with the processes outlined for assessing study eligibility.

The Grading of Recommendations, Assessment, Development and Evaluation (GRADE) framework was used to assess the certainty of the evidence.^7^ The tool was used to apply an overall rating to the body of evidence for each outcome of interest. The major domains of GRADE are risk of bias, imprecision, inconsistency, indirectness, and publication bias. Evidence is graded as being of very low, low, moderate, or high certainty. Evidence from observational data is graded starting at the low-certainty level in the GRADE framework.

### Statistical analysis

We conducted meta-analyses of observational studies with multivariable adjusted data. We estimated the pooled risk ratios (RRs) and their corresponding 95% confidence intervals (CIs) using the inverse variance method. As only a small number of studies were included in each of the main analyses, we chose to use fixed-effects models.^8^ Between-study statistical heterogeneity was quantified using the I^2^ value, and heterogeneity was considered to be important when I^2^ was greater than 30%.^9^

Absolute risk differences (ARDs) and their corresponding 95% CIs were calculated for short-term and long-term outcomes using methods described in the Cochrane Handbook for Systematic Reviews of Interventions.^10^ For each outcome, we estimated the baseline short-term and long-term absolute risks of events using the most representative data available from patients with POAF. We used the pooled RRs to estimate the ARD. We then estimated the absolute risk of each outcome and its corresponding 95% CIs by adding the ARD and its corresponding 95% CIs to the baseline risk estimate.

We planned several analyses *a priori* to identify potential sources of heterogeneity. We performed subgroup analyses based on the following: follow-up length (short-term vs long-term); type of surgery performed (coronary artery bypass surgery [CABG] ± other procedures vs isolated valvular surgery); and type of anticoagulation therapy used (patients using either a non-vitamin K antagonist oral anticoagulant [NOAC] or vitamin K antagonist [VKA] vs VKA alone). To assess the robustness of our findings, we performed sensitivity analyses of studies that excluded patients with preoperative AF, studies that were at moderate or low risk of bias, and studies with and without multivariable adjusted data. For the outcome of arterial thromboembolism, we performed a sensitivity analysis excluding studies that defined arterial thromboembolism as total stroke (ie, included nonischemic strokes).

One study compared patients using VKAs and patients using NOACs against the same comparator group in 2 separate analyses.^11^ To avoid duplication of the comparator group, we included only patients using VKAs for the main analyses in the current study. We then conducted sensitivity analyses with only patients using NOACs.

Methods for the analysis of unpublished data obtained from the Coronary Artery Bypass Grafting Surgery Off- or On-pump Revascularisation Study (CORONARY) trial are detailed in the Supplemental Methods S2. All analyses were conducted using Review Manager (Cochrane Collaboration, London, UK), version 5.4. Analyses were 2-tailed, with statistical significance set at *P* < 0.05.

## Results

### Study selection

We identified 14,988 unique citations. After reviewing the full text of 167 articles, we found that 9 observational studies met the eligibility criteria.^11^, ^12^, ^13^, ^14^, ^15^, ^16^, ^17^, ^18^, ^19^ No randomized controlled trials were identified. A flow diagram of the study selection process is shown in Supplemental Figure S1. Of the 254,200 participants with POAF included, 27.1% were prescribed anticoagulation therapy after surgery. Mortality was reported in 7 studies, arterial thromboembolism in 6 studies, and bleeding, myocardial infarction, and venous thromboembolism in 2 studies each. Details regarding the selection of specific studies are provided in the Supplemental Results S1

### Study characteristics

The characteristics of the 9 included studies are outlined in Table 1. The mean (standard deviation) participant age was 68.4 years (9.0), and 22% were female. Studies included patients undergoing either isolated CABG surgery (6 studies), CABG surgery with or without a concomitant procedure (2 studies), or isolated valvular surgery (1 study). The average short-term and long-term follow-up time was 30 days (range: 30-90; 3 studies) and 5.0 years (range: 4.2-10.0; 6 studies), respectively.

**Table 1:** Baseline characteristics of included studies

Author	Year	Country	Surgical subtype	Surgery, N	POAF, N (%)	POAF definition	Discharge medications		CHADS_2_ Score AC/no AC	CHA_2_DS₂-VASc Score AC / no AC	Follow-up period
							AC use, %	ASA use, % AC / no AC			
Butt^13^	2018	Denmark	Isolated CABG	7524	2108 (28)	AF requiring either medical therapy or cardioversion	—	61	1.3	3.1	5.1 y
Butt^12^	2019	Denmark	Isolated valvular	1587	675 (43)	AF requiring either medical therapy or cardioversion	—	40	1.4	2.9	4.2 y
CORONARY^16^	—	International	Isolated CABG	4752	687 (14)	AF lasting > 5 min and requiring treatment	10	96	—	—	4.7 y
Matos^17^	2021	US, Canada	Isolated CABG	1,075,433	166,946 (16)	AF lasting > 1 h and/or requiring treatment	24	—	—	3.3 / 3.1	30 d
El-Chami^14^	2010	US	CABG ± other	16,169	2985 (18)	AF lasting > 1 h and/or requiring treatment	21	86	—	—	6 y
Hata^15^	2013	Japan	Isolated CABG	447	151 (34)	AF requiring defibrillation by intravenous medication or electrical cardioversion	38	100	2.6 / 2.2	—	3 mo
Marazzato^19^	2021	Italy	Isolated CABG	665	208 (31)	Detected by 12-lead electrocardiogram during routine clinical assessment	—	—	—	—	10 y
Nauffal^11^	2021	US	CABG ± valve, isolated valvular	—	73,072	AF lasting > 1 h and/or requiring treatment	36	92 / 97	—	4.0 / 3.9	30 d
Taha^18^	2021	Sweden	Isolated CABG	24,523	7368 (30)	New AF diagnosis during index hospitalization or within 30 d using Swedish National Patient Registry, or cardioversion during index hospitalization	24	76 / 98∗	—	—	4.5 y

AC, anticoagulation; AF, atrial fibrillation; ASA, acetylsalicylic acid; CABG, coronary artery bypass graft; CHADS_2_ score – **C**ongestive heart failure, **H**ypertension, **A**ge, **D**iabetes, **S**troke/thromboembolism; CHA_2_DS_2_-VASc score, **C**ongestive heart failure, **H**ypertension, **A**ge, **D**iabetes, **S**troke/thromboembolism, **V**ascular disease, **S**ex (female); CORONARY, Coronary Artery Bypass Grafting Surgery Off- or On-pump Revascularisation Study; POAF, perioperative atrial fibrillation.

∗ Any antiplatelet drug use, including ASA.

The diagnosis of POAF was made based on database records in 6 studies, by prospective data collection as part of a randomized trial in 1 study, by physician interview and chart review in 1 study, and by an unspecified review of medical records in 1 study. The POAF definitions used in individual studies are summarized in Table 1. Eight studies excluded patients with preoperative AF. Anticoagulation therapy use was defined as the use of a VKA in 6 studies, and use of either an NOAC or a VKA in 3 studies. Anticoagulation status after surgery was determined by medication use at hospital discharge in 5 studies, medication use within 30 days after discharge in 3 studies, and new in-hospital medication use in 1 study. Three of the 8 studies reporting long-term outcomes assessed anticoagulation status on subsequent follow-up. Two studies assessed anticoagulation status over the entire follow-up duration,^12^^,^^13^ and 1 study assessed anticoagulation status for up to 1 year after surgery.^16^

For the outcome of arterial thromboembolism, 3 studies reported a composite of ischemic stroke and systemic embolism, 2 studies reported ischemic stroke, and 2 studies reported total stroke. The diagnosis of arterial thromboembolism was based on database records in 5 studies, by prospective data collection during a randomized trial in 1 study, and by an unspecified review of medical records in 1 study. For the outcome of bleeding, 1 study reported hospital admissions for bleeding, and 1 study reported major bleeding events. Both studies made the diagnosis of bleeding by using database records.

### Risk of bias and certainty of evidence

For arterial thromboembolism, the risk of bias was moderate for 3 studies, serious for 1 study, and critical for 2 studies. For bleeding, the risk of bias was serious for 1 study and critical for 1 study. The assessments for risk of bias conducted in individual studies are detailed in Supplemental Figure S2. The certainty-of-evidence assessments for individual outcomes are detailed in Supplemental Table S1.

### Risk of arterial thromboembolism

**Figure 1:**
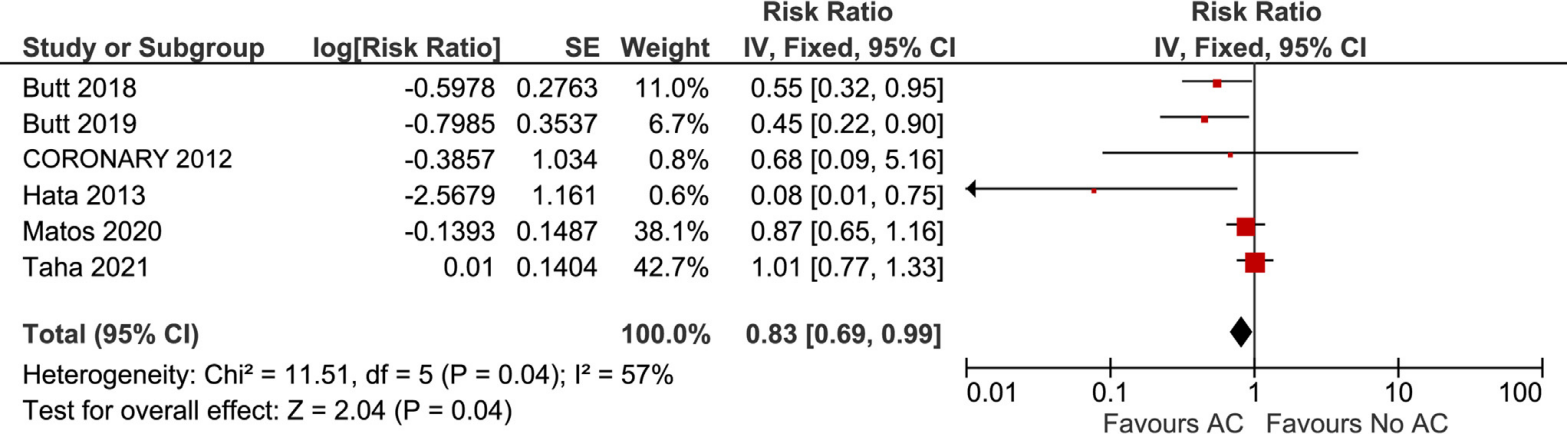
Forest plot for risk of arterial thromboembolism. AC, anticoagulation therapy; CI, confidence interval; CORONARY, Coronary Artery Bypass Grafting Surgery Off- or On-pump Revascularisation Study; df, degrees of freedom; IV, inverse variance; SE, standard error.

The relative risk of arterial thromboembolism was significantly lower in patients using anticoagulation therapy (RR 0.83; 95% CI, 0.69-0.99; *P* = 0.04; I^2^ = 57%; 6 studies; 177,241 patients; Fig. 1). Of the 4 studies that reported long-term outcomes, 3 accounted for anticoagulation therapy use on subsequent follow-up.^12^^,^^13^^,^^16^ The estimated short-term incidence of arterial thromboembolism was 3.7% in patients using anticoagulation therapy, and 4.5% in patients not receiving anticoagulation therapy (ARD -0.8; 95% CI, -1.4 to -0.4). The estimated long-term incidence of arterial thromboembolism was 9 per 1000 person-years in patients receiving anticoagulation therapy, and 11 per 1000 person-years in patients not receiving anticoagulation therapy (ARD -2; 95% CI, -4 to 0). The certainty of the evidence grade for short-term and long-term outcomes was very low and low, respectively (Table 2). The certainty was downgraded for concerns related to risk of bias and indirectness of the evidence.

**Table 2:** Summary of study results

Outcome	Participants, n; studies, n	Relative effect (95% CI)	Follow-up period	Anticipated absolute effects in study population (95% CI)			Certainty of evidence grade
				Risk without AC	Risk with AC	Difference	
Arterial thromboembolism	177,241;6	0.83 (0.69–0.99)	ST	4.5	3.7 (3.1 to 4.1)	–0.8 (–1.4 to –0.4)	VL
			LT	11	9 (8 to 11)	–2 (–4 to 0)	L
Bleeding	173,620;2	3.22 (2.82–3.68)	ST	0.2	0.7 (0.6 to 0.8)	0.5 (0.4 to 0.6)	VL
			LT	19	61 (54 to 70)	42 (35 to 51)	VL
All-cause mortality	180,283;7	1.00 (0.91–1.09)	ST	0.4	0	0	VL
			LT	29	29 (26 to 32)	0 (–3 to 3)	VL
Myocardial infarction	57,006;2	0.67 (0.44–1.02)	ST	3.8	2.6 (1.7 to 3.9)	–1.2 (–2.1 to 0.1)	VL
			LT	4	3 (2 to 4)	–1 (–1 to 0)	VL
Venous thromboembolism	63,552;2	0.42 (0.29–0.61)	ST	0.5	0.2 (0.1 to 0.3)	–0.3 (–0.4 to –0.2)	L
			LT	3	1 (1 to 2)	–2 (–2 to -1)	L

Risk without anticoagulation therapy (AC), risk with AC, and difference are given as % for short-term (ST), and per 1000 person-years for long-term (LT).

CI, confidence interval; L, low; VL, very low.

### Risk of bleeding

**Figure 2:**
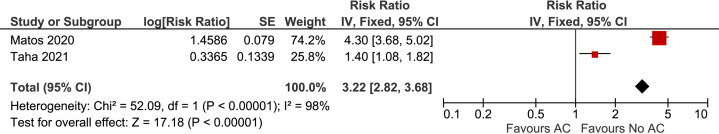
Forest plot for risk of bleeding. AC, anticoagulation therapy; CI, confidence interval; df, degrees of freedom; IV, inverse variance; SE, standard error.

The relative risk of bleeding was significantly higher in patients using anticoagulation therapy (RR 3.22; 95% CI, 2.82-3.68, I^2^ = 98%; *P* < 0.001; 2 studies; 173,620 participants; Fig. 2). The estimated short-term incidence of bleeding was 0.7% in patients receiving anticoagulation therapy use and 0.2% in patients not receiving anticoagulation therapy (ARD 0.5; 95% CI, 0.4 to 0.6). The estimated long-term incidence of bleeding was 61 and 19 per 1000 person-years in patients receiving vs not receiving anticoagulation therapy (ARD 42; 95% CI, 35 to 51), respectively. The grade of certainty of the evidence was very low (Table 2). The certainty was downgraded for concerns related to risk of bias, indirectness of the evidence, and inconsistency in the reported findings.

### Other outcomes

The relative risk of venous thromboembolism was significantly lower in patients receiving anticoagulation therapy (RR 0.42; 95% CI, 0.29-0.61, I^2^ = 0%; 2 studies; 63,552 patients; low certainty of evidence; Supplemental Figure S3). Use of anticoagulation therapy had no significant effects on the risk of mortality (RR 1.00; 95% CI, 0.91-1.09, I^2^ = 85%; 7 studies; 180,283 participants; very low certainty of evidence; Supplemental Figure S4) or myocardial infarction (RR 0.67; 95% CI, 0.44-1.02, I^2^ = 85%; n = 56,319; 2 studies; very low certainty of evidence; Supplemental Figure S5).

### Subgroup analyses

A significant interaction was observed between mortality and the length of follow-up (*P* for interaction = 0.005). However, only a single study reported mortality outcomes after ≤ 3 months of follow-up.^17^ Other subgroup analyses did not yield significant results (Supplemental Table S2).

### Sensitivity analyses

For the outcome of arterial thromboembolism, a sensitivity analysis excluding studies at high and critical risk of bias revealed a lower relative risk when compared to the main results (RR 0.52; 95% CI, 0.34-0.78; 3 studies; 3470 participants). Other preplanned sensitivity analyses did not indicate any meaningful differences in results when compared to the main findings (Supplemental Table S3).

## Discussion

In this systematic review and meta-analysis of patients with POAF after cardiac surgery, we found observational data suggesting that anticoagulation therapy is associated with a 17% lower risk of arterial thromboembolism but also a 3-fold increased risk of bleeding. No randomized controlled trials were identified. Although these findings suggest that anticoagulation therapy could be effective for stroke prevention in this population, no high-quality evidence is currently available to inform this issue.

Prescribing practices for anticoagulation therapy after POAF vary widely among clinical practitioners, with the use of anticoagulation therapy at hospital discharge having been reported to be anywhere between 3.6% and 50%,^1^ which may reflect the lack of high-quality randomized data available to inform clinical practice. In addition, current guidelines vary in their recommendations on how anticoagulation therapy should be prescribed in this setting. The American College of Cardiology and American Heart Association recommend that anticoagulation therapy be prescribed using the same considerations as for nonsurgical AF,^20^ and the European Society of Cardiology suggests that long-term anticoagulation therapy should be considered in all patients who are at increased risk of stroke.^21^ By comparison, the Canadian Cardiovascular Society recommends that any anticoagulation therapy prescribed for POAF should be reconsidered 6 to 12 weeks after initiation, due to insufficient evidence available for recommending its long-term use.^22^ Our study found that anticoagulation therapy was associated with a small reduction in the long-term absolute risk of arterial thromboembolism, and that this potential benefit may be outweighed by the higher bleeding risk. However, the methodologic limitations of individual studies should be considered carefully when interpreting our findings. For example, the only observational study that assessed both long-term risk of arterial thromboembolism and bleeding found that use of anticoagulation therapy within 30 days of hospital discharge was associated with an increased risk of major bleeding (adjusted hazard ratio 1.40; 95% CI, 1.08-1.82), with no difference in the risk of arterial thromboembolism (adjusted hazard ratio 1.01; 95% CI, 0.77-1.33) after 5 years of follow-up.^18^ However, drug discontinuations were not accounted for in this study. Given that patients with POAF often have their anticoagulation therapy discontinued over time,^23^ the efficacy of anticoagulation therapy use may have been underestimated. In addition, the risk of major bleeding may have been overestimated, as many diagnoses of uncertain clinical significance (eg, hematuria, epistaxis, iron deficiency anemia) were included in the outcome definition. Higher-quality studies are needed to better characterize stroke risk in patients with and without anticoagulation therapy, before anticoagulation therapy can be considered routinely in clinical practice.

Our study found that a short course of anticoagulation therapy was associated with a 0.8% absolute reduction in the risk of arterial thromboembolism and a 0.5% absolute increase in the risk of bleeding. Although these findings appear to suggest that the benefits associated with short-term anticoagulation therapy after POAF may be slightly greater than the associated risks, the certainty in the available evidence is low. In fact, results from the largest study we identified in our review did not support the routine use of short-term anticoagulation therapy after POAF.^17^ In this study of more than 160,000 POAF patients registered in the Society of Thoracic Surgeons database, anticoagulation therapy use on discharge was associated with an increase in the 30-day risk of hospital readmissions for bleeding (adjusted odds ratio 4.30; 95% CI, 3.69-5.02; ARD 0.75%), without any difference in readmissions for stroke (adjusted odds ratio 0.87; 95% CI, 0.65-1.16; ARD 0.01%). However, as this study had a significantly lower incidence of stroke than that previously reported,^1^ the absolute risk reduction may have been underestimated. Findings from the ongoing Anticoagulation for New-Onset Post-Operative Atrial Fibrillation After CABG (PACES) trial will provide higher-quality evidence on whether short-term anticoagulation therapy after POAF is a safe and effective strategy for thromboembolic prevention in CABG patients.^24^ In the meantime, clinicians should continue to weigh carefully the potential risks and benefits of use of anticoagulation therapy for each individual patient.

Our study demonstrated that anticoagulation therapy was associated with a relative-risk reduction of 17% for the outcome of arterial thromboembolism. By comparison, the relative-risk reduction achieved using VKAs and NOACs among patients with clinical AF ranges between 62% and 73%.^25^ Differences in the underlying pathophysiology of stroke may explain these discrepancies. POAF has been hypothesized to be a marker of increased vascular disease burden, as these 2 entities share many of the same underlying risk factors.^26^ Patients with POAF may therefore be at increased risk of ischemic stroke from small-vessel occlusions and large-artery atherosclerosis. In one study of 576 CABG patients with POAF, large-artery atherosclerosis was responsible for 2 of 4 postoperative strokes.^27^ Oral anticoagulation therapy would not have been effective for preventing these events. Alternatively, anticoagulation therapy might benefit only a subset of POAF patients who develop future episodes of AF. A systematic review of 8 studies found that the incidence of POAF recurrence identified through noninvasive monitoring within the first 4 weeks after hospital discharge was 28.3% (95% CI, 23.0-33.6).^28^ One possibility is that patients who do not experience AF recurrence are at a lower risk of long-term stroke, and therefore derive less benefit from anticoagulation therapy. Whether anticoagulation therapy is warranted in POAF patients who usually have a low AF burden remains unclear. Results from the Implantable Loop Recorder Detection of Atrial Fibrillation to Prevent Stroke (LOOP) trial suggest that use of anticoagulation therapy in patients with subclinical, low-burden AF may lead to a much smaller relative-risk reduction than it does in patients with clinical AF. This study of 6205 elderly patients randomized participants from the general population to either an implantable loop recorder or usual care, with episodes of AF lasting 6 minutes or greater on a loop recorder being treated with anticoagulation therapy. Although 31.8% of patients with loop recorders were subsequently found to have AF, no significant difference was seen in the time to first stroke or systemic arterial embolism after a median follow-up time of 5.4 years (hazard ratio 0.80; 95% CI, 0.61-1.05).^29^ Whether high-risk subsets of POAF patients derive greater benefit from anticoagulation therapy is currently unknown. Although clinicians tend to prescribe anticoagulation therapy more frequently in patients who have prolonged episodes of AF or a higher number of traditional stroke risk factors, such strategies are unproven.^30^

Our systematic review has limitations. The certainty of the evidence was found to be low to very low across outcomes, owing to a substantial degree of heterogeneity, a low number of studies, and a high risk of bias. The summary risk ratios should therefore be interpreted with caution. Bleeding definitions were heterogeneous across studies, with 1 study having included events of uncertain clinical importance. Consequently, the precise risk of major bleeding in these patients cannot be determined with certainty. Most studies included only patients undergoing CABG surgery. Whether our findings pertain to patients undergoing isolated valvular surgery is unclear. Some of the studies reporting long-term outcomes did not assess whether anticoagulation was continued after hospital discharge, which may have underestimated our relative-risk estimates. We assumed the presence of a shared relative-risk estimate between short-term and long-term outcomes. Although no significant differences were identified between short-term and long-term subgroups for the main study outcomes, the import of these analyses is limited by the low number of studies.

## Conclusion

In this systematic review and meta-analysis of patients with POAF after cardiac surgery, use of anticoagulation therapy was associated with a reduced risk of arterial thromboembolism but also an increased risk of bleeding. However, only observational studies were identified, and the grade of certainty of the evidence was low to very low. Randomized controlled trials are needed to address this important issue.

## Footnotes

To access the supplementary material accompanying this article, visit *CJC Open* at https://www.cjcopen.ca/ and at https://doi.org/10.1016/j.cjco.2022.06.003.
