# Supplementary material for: Use of Anticoagulation Therapy in Patients With Perioperative Atrial Fibrillation After Cardiac Surgery: A Systematic Review and Meta-analysis
Source: CJC Open. 2022 Jun 10;4(10):840–7. doi: 10.1016/j.cjco.2022.06.003 (PMC9568684; doi:10.1016/j.cjco.2022.06.003)
Supplement: Supplementary Material [file mmc1.pdf]

Supplemental Files

Supplemental Methods S1. EMBASE search strategy.

Supplemental Methods S2. Methods for analysis of unpublished data from the CORONARY trial.

Supplemental Results. Additional study selection results.

Supplemental Figure S1. PRISMA flow chart.

Supplemental Figure S2. Risk of bias assessment using the ROBINS-I tool.

Supplemental Figure S3. Meta-analysis for venous thromboembolism.

Supplemental Figure S4. Meta-analysis for mortality.

Supplemental Figure S5. Meta-analysis for myocardial infarction.

Supplemental Table S1. Summary of GRADE assessment.

Supplemental Table S2. Subgroup analyses for arterial thromboembolism and mortality.

Supplemental Table S3. Sensitivity analyses.

Supplemental Methods S1. EMBASE search strategy.

1. exp atrial fibrillation/
2. atrial fibrillation.mp.
3. poaf.mp.
4. af.mp.
5. afib.mp.
6. 1 or 2 or 3 or 4 or 5
7. exp surgery/
8. exp perioperative period/
9. exp postoperative care/
10. exp postoperative complication/
11. surg*.mp.
12. periop*.mp.
13. postop*.mp.
14. operati*.mp.
15. 7 or 8 or 9 or 10 or 11 or 12 or 13 or 14
16. exp anticoagulation/
17. exp anticoagulant agent/
18. anticoag*.mp.
19. antithromb*.mp.
20. noac.mp.
21. oac.mp.
22. warfarin.mp.
23. edoxaban.mp.
24. rivaroxaban.mp.
25. apixaban.mp.
26. dabigatran.mp.
27. lmwh.mp.
28. ufh.mp.
29. heparin.mp.
30. dalteparin.mp.
31. enoxaparin.mp.
32. tinzaparin.mp.
33. nadroparin.mp.
34. 16 or 17 or 18 or 19 or 20 or 21 or 22 or 23 or 24 or 25 or 26 or 27 or 28 or 29 or 30 or 31 or 32 or 33
35. 6 AND 15 AND 34
36. animals/ NOT (humans/ AND animals/)
37. meeting abstract.pt. or conference abstract.pt. or meta-analysis.pt. or review.pt. or systematic review.pt. or editorial.pt. or comment.pt. or case reports.pt.
38. 35 NOT 36 NOT 37

Supplemental Methods S2. Methods for analysis of unpublished data from the CORONARY trial.

Data Source

This is a retrospective secondary analysis of the CORONARY randomized controlled trial. The study methods, study results, and associations between perioperative atrial fibrillation (POAF) and long-term outcomes have been described elsewhere.[1, 2, 3] In brief, 4752 adult patients across 19 countries were pre-operatively randomized to on-pump versus off-pump coronary artery bypass surgery (CABG) with a primary composite outcome of death, nonfatal stroke, nonfatal myocardial infarction, or new renal failure requiring dialysis. Research assistants recorded baseline co-morbidities upon randomization. Patients were followed by research assistants during the index hospitalization and assessed during follow-up visits at 30 days, 6 months, 1 year, and yearly thereafter post-randomization. An additional final visit was performed at the end of the study.

Study Population

Adult patients were enrolled into CORONARY if they required isolated CABG with median sternotomy and had additional risk factors. Patients enrolled in CORONARY were included in the current analysis if all the following criteria were met: (1) Documented POAF within 30 days after surgery; (2) no history of pre-operative AF; (3) survived until discharge; (4) no warfarin use at randomization. The index date was defined as the discharge date for the index hospitalization.

Exposure, Outcomes, and Post-Discharge Oral Anticoagulation Use

POAF events were documented by research assistants during the index hospitalization. Stroke was documented during the index hospitalization and on each follow-up visit. Warfarin use was documented on the randomization, discharge, 30-day, and 1-year follow-up visits. Strokes (i.e., a composite of ischemic stroke, transient ischemia attack [TIA], and hemorrhagic stroke) were reviewed by a blinded event adjudication committee.

Statistical Analysis

Analyses were conducted using a complete case analysis approach. Patients with missing covariate data or who were lost to follow-up were excluded from the main analysis. Patients were censored at the time of stroke, time of death, or when the study end date was reached.

Cox regression analysis was used to estimate adjusted hazard ratios with 95% confidence intervals for the association between warfarin use and stroke, adjusted for age and CHA_2DS_2-VASc score. Age and CHA_2DS_2-VASc score co-variates were forced into the model. Warfarin use was treated as a time-dependent covariate. As effect modification between co-variates was not clinically anticipated, interaction terms were not tested in the model. Ties were handled using the Breslow approximation. The proportional hazards assumption was tested by assessing the statistical significance the Schoenfeld residuals test.

Statistical analyses were conducted using STATA 16.0 (StataCorp LLC). All analyses were performed on a 2-sided significance level of 0.05.

<u>References</u>

1. Lamy A, Devereaux PJ, Prabhakaran D, Taggart DP, Hu S, Paolasso E, et al. Off-pump or on-pump coronary-artery bypass grafting at 30 days. N Engl J Med. 2012;366(16):1489-97.
2. Lamy A, Devereaux PJ, Prabhakaran D, Taggart DP, Hu S, Straka Z, et al. Five-Year Outcomes after Off-Pump or On-Pump Coronary-Artery Bypass Grafting. N Engl J Med. 2016;375(24):2359-68.
3. Conen D, Wang MK, Devereaux PJ, Whitlock R, McIntyre WF, Healey JS, et al. New-onset perioperative atrial fibrillation after coronary artery bypass grafting and long-term risk of adverse events – An analysis from the CORONARY trial. Journal of the American Heart Association. 2021;10(12):e020426.

Supplemental Results S1. Additional study selection results.

We contacted study authors for unpublished data and clarifications on reported data. We received clarifications on the number of participants receiving anticoagulation from two study authors[1-3] and the use of multivariable adjustment from one study author.[4] We obtained original study data from the primary author of the CORONARY randomized controlled trial, and conducted multivariable Cox regression analyses using these data (Supplement 4).[5]

Two studies analyzed data from the Society of Thoracic Surgeons database and reported results for the outcomes of thromboembolism, mortality, and bleeding with overlapping participants.[4, 6] We chose to only use data from the larger study for the main analyses.[6]

References

1. Butt JH, Olesen JB, Gundlund A, Kumler T, Olsen PS, Havers-Borgersen E, et al. Long-term Thromboembolic Risk in Patients With Postoperative Atrial Fibrillation After Left-Sided Heart Valve Surgery. JAMA Cardiol. 2019;4(11):1139-47.
2. Butt JH, Xian Y, Peterson ED, Olsen PS, Rorth R, Gundlund A, et al. Long-term Thromboembolic Risk in Patients With Postoperative Atrial Fibrillation After Coronary Artery Bypass Graft Surgery and Patients With Nonvalvular Atrial Fibrillation. JAMA Cardiol. 2018;3(5):417-24.
3. Woldendorp K, Khadra S, Bannon PG, Robinson BM. Novel Oral Anticoagulants Compared to Warfarin for Postoperative Atrial Fibrillation After Isolated Coronary Artery Bypass Grafting. Heart Lung Circ. 2020;29(12):1832-8.
4. Nauffal V, Trinquart L, Osho A, Sundt TM, Lubitz SA, Ellinor PT. Non-Vitamin K Antagonist Oral Anticoagulant vs Warfarin for Post Cardiac Surgery Atrial Fibrillation. Ann Thorac Surg. 2021;112(5):1392-401.
5. Lamy A, Devereaux PJ, Prabhakaran D, Taggart DP, Hu S, Paolasso E, et al. Off-Pump or On-Pump Coronary-Artery Bypass Grafting at 30 Days. New England Journal of Medicine. 2012;366(16):1489-97.
6. Matos JD, McIlvaine S, Grau-Sepulveda M, Jawitz OK, Brennan JM, Khabbaz KR, et al. Anticoagulation and amiodarone for new atrial fibrillation after coronary artery bypass grafting: Prescription patterns and 30-day outcomes in the United States and Canada. J Thorac Cardiovasc Surg. 2021;162(2):616-24 e3.

Supplemental Figure S1. PRISMA flow chart.

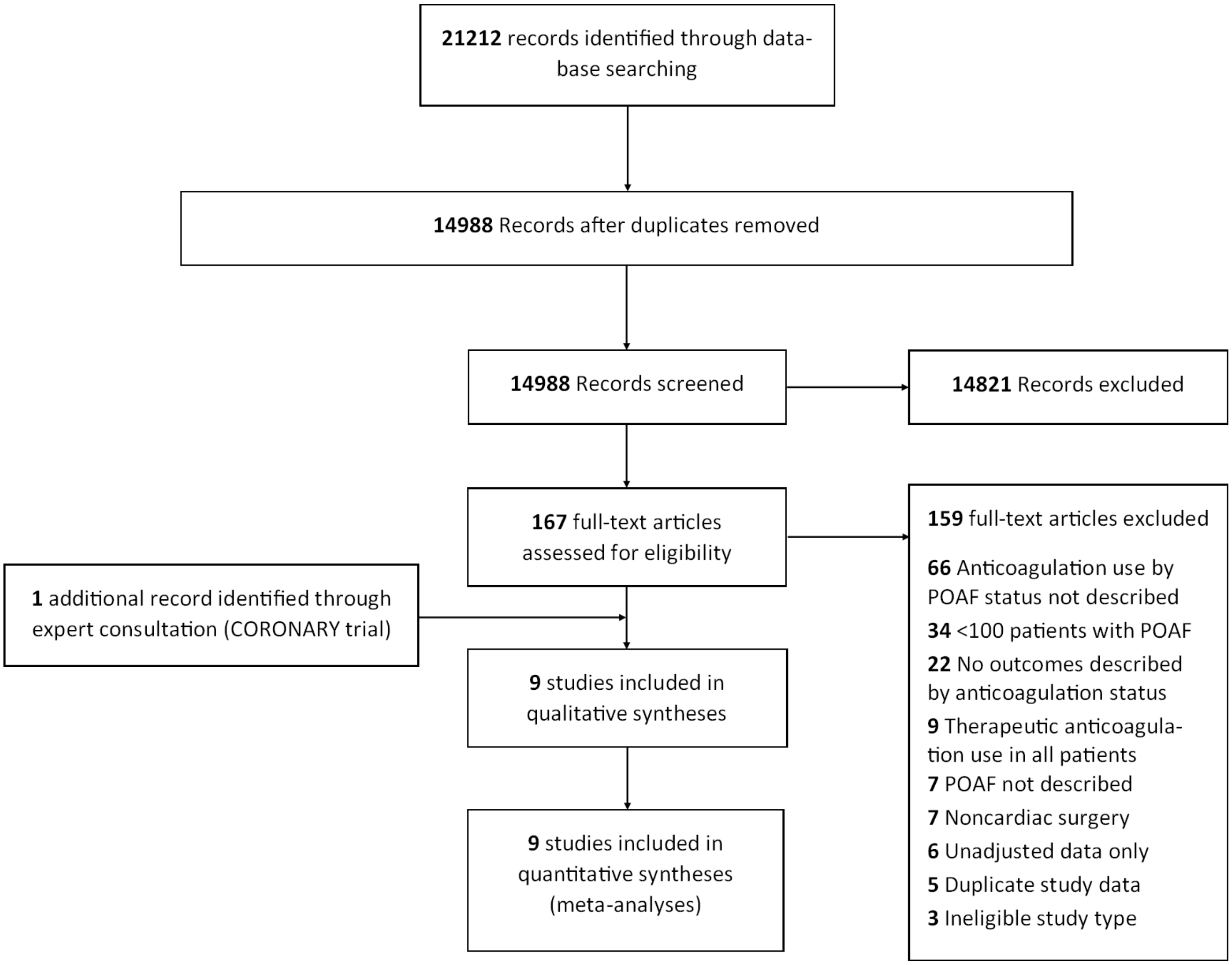

Supplemental Figure S2. Risk of bias assessment using the ROBINS-I tool.

a. Risk of Bias Legend

Low	Low
Moderate	Moderate
Serious	Serious
Critical	Critical
No Information	No Information

b. Arterial thromboembolism

	Confounding	Participant Selection	Classification of Interventions	Deviations from Interventions	Missing Data	Measurement of Outcomes	Selection of Reported Results	Overall
Butt 2018	Moderate	Low	Low	Low	No Information	Low	Low	Moderate
Butt 2019	Moderate	Low	Low	Low	No Information	Low	Low	Moderate
CORONARY	Moderate	Low	Low	Low	Low	Low	Low	Moderate
Hata 2013	Serious	Serious	Low	Low	No Information	Low	Low	Critical
Matos 2020	Serious	Low	Low	Low	Moderate	Low	Moderate	Critical
Taha 2021	Serious	Low	Low	Low	No Information	Low	Low	Serious

c. Bleeding

	Confounding	Participant Selection	Classification of Interventions	Deviations from Interventions	Missing Data	Measurement of Outcomes	Selection of Reported Results	Overall
Matos 2020	Orange	Green	Green	Green	Yellow	Green	Yellow	Red
Taha 2021	Orange	Green	Green	Green	Grey	Green	Green	Orange

d. Mortality

	Confounding	Participant Selection	Classification of Interventions	Deviations from Interventions	Missing Data	Measurement of Outcomes	Selection of Reported Results	Overall
Butt 2018	Yellow	Green	Green	Green	Grey	Green	Green	Yellow
Butt 2019	Yellow	Green	Green	Green	Grey	Green	Green	Yellow
CORONARY	Orange	Green	Green	Green	Green	Green	Green	Orange
El-Chami 2010	Yellow	Green	Green	Orange	Green	Green	Green	Orange
Marazzato 2021	Orange	Green	Green	Green	Grey	Green	Yellow	Orange
Matos 2020	Orange	Green	Green	Green	Yellow	Green	Yellow	Red
Taha 2021	Orange	Green	Green	Green	Grey	Green	Green	Orange

e. Venous thromboembolism

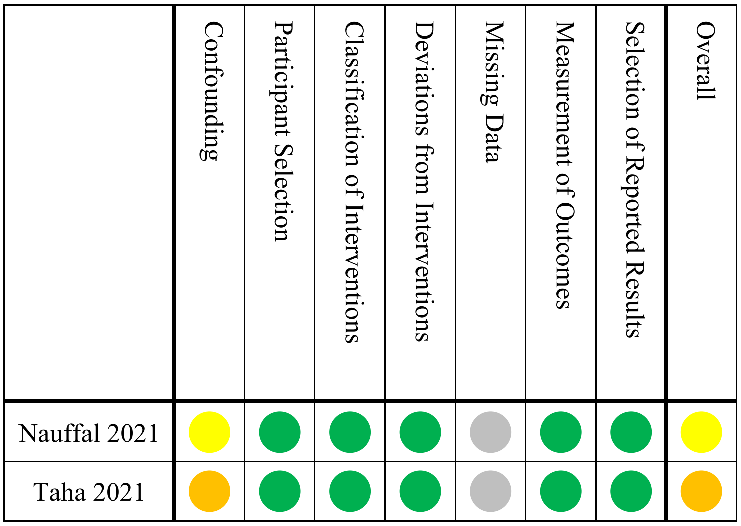

f. Myocardial Infarction

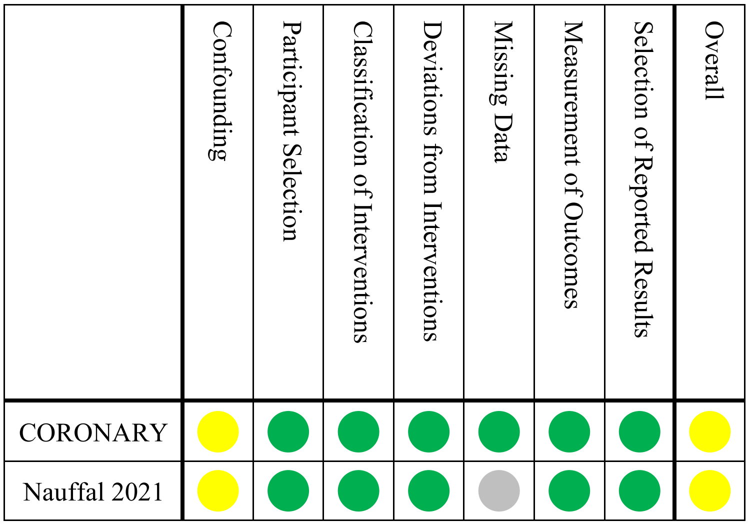

Supplemental Figure S3. Meta-analysis for venous thromboembolism.

Study or Subgroup	log[Risk Ratio]	SE	Weight	Risk Ratio IV, Fixed, 95% CI
Nauffal 2021 [NOAC]	-0.9943	0.2467		Not estimable
Nauffal 2021 [VKA]	-0.9163	0.2153	78.9%	0.40 [0.26, 0.61]
Taha 2021	-0.6931	0.416	21.1%	0.50 [0.22, 1.13]
**Total (95% CI)**			**100.0%**	**0.42 [0.29, 0.61]**

Heterogeneity: Chi² = 0.23, df = 1 (P = 0.63); I² = 0%
Test for overall effect: Z = 4.55 (P < 0.00001)

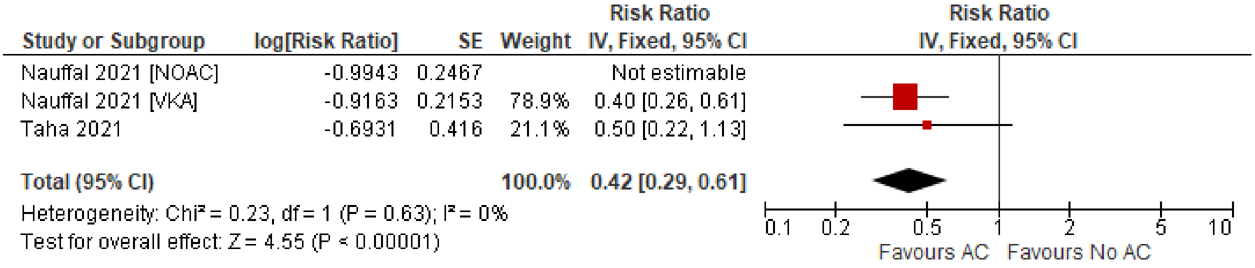

AC – anticoagulation.

Supplemental Figure S4. Meta-analysis for mortality.

Study or Subgroup	log[Risk Ratio]	SE	Weight	Risk Ratio IV, Fixed, 95% CI
Butt 2018	0.0862	0.1452	9.6%	1.09 [0.82, 1.45]
Butt 2019	-0.4463	0.2226	4.1%	0.64 [0.41, 0.99]
CORONARY	1.1184	0.2626	2.9%	3.06 [1.83, 5.12]
El-Chami 2010	-0.2485	0.0842	28.7%	0.78 [0.66, 0.92]
Marazzato 2021	0.4886	0.328	1.9%	1.63 [0.86, 3.10]
Matos 2020	0.1823	0.0786	32.9%	1.20 [1.03, 1.40]
Taha 2021	-0.1165	0.1011	19.9%	0.89 [0.73, 1.09]
**Total (95% CI)**			**100.0%**	**1.00 [0.91, 1.09]**

Heterogeneity: $Chi^2 = 40.14$, $df = 6$ ($P < 0.00001$); $I^2 = 85\%$
Test for overall effect: $Z = 0.05$ ($P = 0.96$)

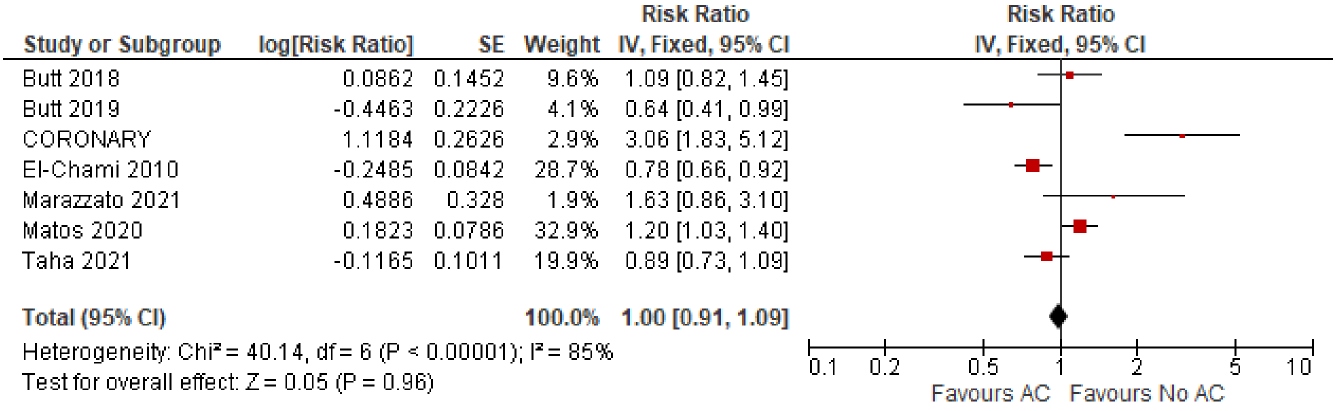

AC – anticoagulation.

Supplemental Figure S5. Meta-analysis for myocardial infarction.

Study or Subgroup	log[Risk Ratio]	SE	Weight	Risk Ratio IV, Fixed, 95% CI
CORONARY	1.2206	0.6666	10.5%	3.39 [0.92, 12.52]
Nauffal 2021 [NOAC]	-0.478	0.2284		Not estimable
Nauffal 2021 [VKA]	-0.5978	0.2281	89.5%	0.55 [0.35, 0.86]
**Total (95% CI)**			**100.0%**	**0.67 [0.44, 1.02]**

Heterogeneity: Chi² = 6.66, df = 1 (P = 0.010); I² = 85%
Test for overall effect: Z = 1.89 (P = 0.06)

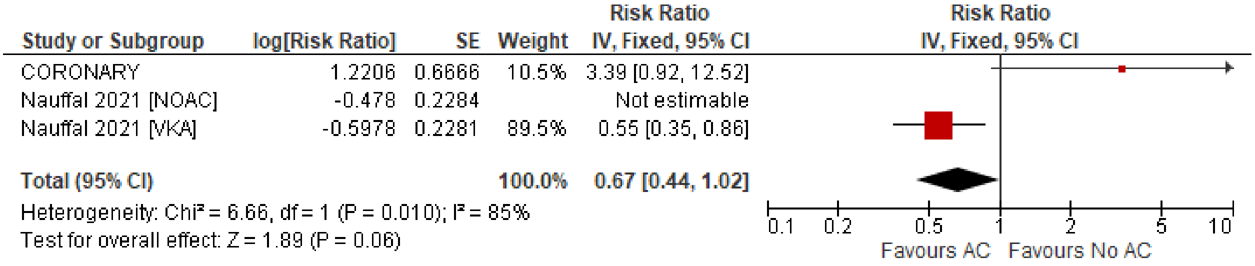

AC – anticoagulation.

Supplemental Table S1. Summary of GRADE assessment.

Outcome	N of participants (studies)	Follow-up Period	Factors that may increase or decrease certainty of evidence				
			Risk of bias	Indirectness	Inconsistency	Imprecision	Other
Arterial Thromboembolism	177241 patients 6 studies	Short-Term	Very serious [a]	Serious [c]	No	No	No
		Long-Term	Very serious [a]	No	No	No	No
Bleeding	173620 patients 2 studies	Short-Term	Very serious [b]	Serious [d]	Very serious [f]	No	Large magnitude of effect [h]
		Long-Term	Very serious [b]	Serious [d]	Very serious [f]	No	Large magnitude of effect [h]
All Cause Mortality	180283 patients 7 studies	Short-Term	Very serious [b]	Serious [c]	Serious [e]	Serious [g]	No
		Long-Term	Very serious [b]	No	Serious [e]	Serious [g]	No
Myocardial Infarction	57006 patients 2 studies	Short-Term	Very serious [b]	Serious [d]	Very Serious [e]	No	No
		Long-Term	Very serious [b]	Serious [d]	Very Serious [e]	No	No
Venous Thromboembolism	63552 patients 2 studies	Short-Term	Very serious [b]	Serious [d]	No	No	Large magnitude of effect [i]
		Long-Term	Very serious [b]	Serious [d]	No	No	Large magnitude of effect [i]

[a] Very serious concerns were raised due to serious or critical risk of confounding bias across studies and the observational nature of the data. Sensitivity analyses excluding studies at highest risk of bias demonstrated findings potentially inconsistent with the primary analysis.

[b] Very serious concerns were raised due to serious or critical risk of confounding bias across studies and the observational nature of the data.

[c] Serious concerns were raised that only one of the studies reported data on short-term outcomes.

[d] Serious concerns were raised that only one study for short-term and one study for long-term outcomes were included.

[e] Serious concerns were raised due to the presence of moderate or moderate-to-high heterogeneity base on the I^2 statistic, significant or borderline significant chi-square test for heterogeneity, variation in the point estimates, and non-overlapping confidence intervals. Subgroup analyses did not demonstrate a cause of the heterogeneity.

[f] Very serious concerns were raised due to a very high degree of heterogeneity demonstrated by the I^2 statistic, the significant chi-square test for heterogeneity, extreme variation in the point estimates, and non-overlapping confidence intervals.

[g] The optimal information size criterion was not met.

[h] The pooled relative risk was greater than 2. The certainty of evidence was therefore rated up for having a large magnitude of effect.

[i] The pooled relative risk was less than 0.5. The certainty of evidence was therefore rated up for having a large magnitude of effect.

Supplemental Table S2. Subgroup analyses for arterial thromboembolism and mortality.

Subgroup	Relative Risk (95% CI) [studies]	
	Arterial thromboembolism	Mortality
Follow-up ≤3 months	0.84 (0.63, 1.12) [2]	1.20 (1.03, 1.40) [1]
Follow-up >3 months	0.82 (0.66, 1.04) [4]	0.91 (0.82, 1.02) [6]
CABG ± concomitant procedure	0.78 (0.72, 1.04) [5]	1.02 (0.93, 1.11) [6]
Isolated valvular	0.45 (0.22, 0.90) [1]	0.64 (0.41, 0.99) [1]
NOAC ± VKA	0.84 (0.70, 1.01)[4]	1.04 (0.93, 1.16) [4]
VKA	0.24 (0.06, 1.18) [2]	0.92 (0.79, 1.07) [3]

CABG – coronary artery bypass graft; NOAC – novel oral anticoagulation; VKA – vitamin K antagonist.

Supplemental Table S3. Sensitivity analyses.

Outcome	Analysis	RR (95% CI) [studies]
Arterial thromboembolism	Excludes studies that include patients with preoperative AF	0.84 (0.71, 1.01) [5]
Arterial thromboembolism	Excludes high/critical risk of bias	0.52 (0.34, 0.78) [3]
Arterial thromboembolism	Excludes studies with that include hemorrhagic strokes	0.81 (0.64, 1.02) [4]
Arterial thromboembolism	Includes studies with unadjusted analyses	0.84 (0.70, 0.99) [11]
Bleeding	Includes studies with unadjusted analyses	3.18 (0.22, 3.42) [3]
Mortality	Excludes studies that include patients with preoperative AF	1.00 (0.91, 1.09) [7]
Mortality	Excludes high/critical risk of bias	0.93 (0.73, 1.18) [2]
Mortality	Includes studies with unadjusted analyses	0.99 (0.91, 1.08) [9]
Myocardial Infarction	Includes only patients using NOACs from Nauffal 2021	0.74 (0.49, 1.13) [2]
Venous thromboembolism	Includes only patients using NOACs from Nauffal 2021	0.40 (0.26, 0.61) [2]

AF – atrial fibrillation; NOAC – novel oral anticoagulant; RR – relative risk.
